# Chlorhexidine Mouthwash for Gingivitis Control in Orthodontic Patients: A Systematic Review and Meta-Analysis

**DOI:** 10.3290/j.ohpd.b3170043

**Published:** 2022-06-28

**Authors:** Ioanna Karamani, Eleni Kalimeri, Kiriaki Seremidi, Sofia Gkourtsogianni, Dimitrios Kloukos

**Affiliations:** a Researcher, Department of Orthodontics and Dentofacial Orthopedics, 251 Hellenic Air Force & VA General Hospital, Athens, Greece. Substantial contribution to study concept and design, data collection, quality assessment, data analysis.; b Lecturer, Department of Paediatric Dentistry, Dental School, University of Athens, Athens, Greece. Substantial contribution to study concept and design, gave final approval of the version to be published.; c Lecturer, Department of Paediatric Dentistry, Dental School, University of Athens, Athens, Greece. Substantial contribution to study concept and design, data collection, quality assessment, data analysis.; d Lecturer, Department of Orthodontics and Dentofacial Orthopedics, 251 Hellenic Air Force & VA General Hospital, Athens, Greece; Department of Orthodontics and Dentofacial Orthopedics, School of Dental Medicine, University of Bern, Switzerland. Substantial contribution to study concept and design, gave final approval of the version to be published.

**Keywords:** chlorexidine mouthwash, gingivitis, orthodontic treatment

## Abstract

**Purpose:**

To summarise the available data on the effects of chlorhexidine (CHX) mouthwash in treating gingivitis during treatment with fixed orthodontic appliances.

**Materials and Methods:**

Multiple electronic databases were searched up to December 7th, 2021. Only randomised controlled trials (RCTs) were eligible for inclusion. The quality of the included RCTs was assessed with the Cochrane risk of bias tool for randomised trials (RoB 2.0). After data extraction and risk of bias assessment, differences were recorded in several oral hygiene indices in time and mean percentage change in those indices using different antimicrobial solutions.

**Results:**

Fourteen studies were deemed eligible for inclusion, reporting on a total of 602 patients with an age range of 11–35 years. The experimental solution was a 0.06%, 0.12%, or 0.2% CHX mouthwash with the control either a placebo mouthwash or a selection from a variety of mouthwashes. Treatment duration varied from 1 day to almost 5 months and the follow-up period varied from 1 min to 5 months. Chlorhexidine mouthrinses led to reduced plaque accumulation and gingival inflammation during orthodontic treatment, while at the same time, some of the control group mouthrinses were deemed equally effective. No statistically significant difference was detected in the meta-analysis between CHX and mouthwashes with propolis/probiotics/herbs in terms of the gingival index at 3 to 4 weeks (mean difference 0.07, 95% CI: -0.18, 0.31, p = 0.59).

**Conclusion:**

Chlorhexidine mouthwash in orthodontic patients successfully controls gingival inflammation and bleeding when compared to untreated controls, but is equally effective as other mouthrinses where various oral health indices are concerned.

Orthodontic treatment aims at achieving a functional and aesthetically acceptable occlusion. In order to be successfully completed, it needs to ensure the integrity of both hard and soft tissues throughout the entire treatment duration. Orthodontic appliances alter the physicochemical conditions of bacterial growth and cause both qualitative and quantitative changes in bacterial colonies.^11^ In addition, orthodontic appliances not only favour the accumulation and retention of food and debris, but also may protect the plaque from the actions of brushing, mastication and salivary flow.

Clinical studies have proven a direct relationship between the number of plaque bacteria and the pathological consequences.^47^ If this number increases significantly and/or if there are alterations in the effectiveness of the defense mechanisms of the host, the balance of the healthy community structure is disturbed and gingivitis develops. The nature of the imbalance and the ease with which balance can be restored depend on the overall composition of the individual’s microbiome and genetics.^28^ Additionally, distinct community structures were found for gingival health, gingivitis, and periodontitis, demonstrating the relationship between gingival tissue status and the subgingival microbiome. Most species associated with periodontitis were infrequently detected in gingival health, but were often detected in gingivitis – albeit in low abundance – which means that gingivitis and periodontitis are a continuum.^1^

The extracellular matrix is a critical element of microbial biofilms. The matrix affords microbial cells protection against chemical and physical factors and hinders the removal of pathogenic dental biofilm. Consequently, targeting the biofilm matrix seems to be a very good way to disrupt bacterial biofilms, such as those in periodontal pockets, and also to enhance the effectiveness of simultaneously applied antimicrobial agents.^27^

Orthodontic bands and brackets can lead to favourable conditions for the accumulation of dental biofilm, an increase of the bacterial load and therefore development of dental plaque.^35,54^ It is particularly difficult to maintain an acceptable hygiene when bands, wires, and ligatures are involved, with authors reporting development of hyperplastic gingiva within 1 to 2 months after placement of appliances.^34^ Even well-informed orthodontic patients with excellent oral hygiene have some inflammatory changes and dental health problems in areas of difficult access and posterior regions of the mouth.^54^

Correct and adequate mechanical plaque control is the most effective and specific way to interrupt supragingival biofilm development.^9^ There is evidence that brushing associated with flossing, when performed adequately and systematically at regular intervals, can prevent and reverse the inflammatory changes that may happen in the gingival tissues.^30,33,37^ Mouthwashes with chemotherapeutic agents may represent an important complement to mechanical methods in periodontal disease prevention and treatment.^6^ Numerous chemical agents have been evaluated for their ability to influence plaque development. When chemical plaque control agents are reviewed, several basic criteria should be discussed, such as the specifity, efficacy, substantivity, safety and stability of the candidate chemical plaque control product.^14,31^

Chlorhexidine (CHX) is the most widely used antiseptic that has yielded beneficial results as a preventive strategy.^39^ It is a bisbiguanide antiseptic, which is a symmetrical molecule consisting of four chlorophenyl rings and two biguanide groups connected by a central hexamethylene bridge. As an antimicrobrial agent, it is effective in vitro against both Gram-positive and Gram-negative bacteria, including aerobes and anaerobes, yeasts and fungi.^14,16,46^ The binding properties of the CHX molecule result in a broad bactericidal and bacteriostatic spectrum and high substantivity (up to 12 h) within the oral cavity.^46^ Because CHX strongly binds to tissues, it is poorly absorbed in the gastrointestinal tract, reducing its systemic toxicity.^24^ No teratogenic alterations have been found following long-term use.^15^

The application of CHX for plaque and gingivitis control goes back to 1970, when the use of 0.2% chlorhexidine gluconate rinses 2x daily proved to prevent plaque accumulation and subsequent gingivitis.^32^ Nowadays, it is considered the gold standard for the chemical control of biofilm.^32^ CHX is safe, stable and, owing to its great substantivity, effective in preventing and controlling plaque formation, breaking up existing plaque, as well as inhibiting and reducing the development of gingivitis and improving symptoms of periodontitis.^22,24,31^ According to a recent clinical study, the combination of manual toothbrush with chlorexidine mouthwash showed significant improvement of plaque index scores and gingival index scores.^43^

The purpose of this systematic review was to present and assess the available evidence for the effects of chlorhexidine mouthwash exclusively and regardless of the type of toothbrush in treating gingivitis during treatment with fixed orthodontic appliances.

## Materials and Methods

### Reporting Format

The latest (2021) Preferred Reporting Items for Systematic Reviews and Meta-Analyses (PRISMA) were adopted throughout the process of the present systematic review.^36,40^

### Population (P), Intervention (I), Comparison (C), Outcomes (O) (PICOS) and Study Design

- **Participants** (Population): Orthodontic patients of any age and sex with fixed orthodontic appliances.
- **Intervention:** Chlorhexidine mouthwash regardless of chlorhexidine content (%)
- **Comparisons:** Any control mouthwash was accepted, including placebo solutions,
- **Outcomes:** Quantitative analysis of plaque indices (PI), and gingival indices (GI), gingival bleeding index (GBI), oral hygiene index-simplified (OHI-S), probing depth (PD), papilla bleeding index, hyperplastic index (HI) and bonded bracket index.

### Study Design

Only randomised clinical trials (RCTs) were considered eligible for inclusion in this review.

#### Follow-up

All observation periods were accepted.

#### Exclusion criteria

- Non-randomised studies, case reports or studies reporting fewer than 5 patients.
- Studies including patients with removable appliances or palatal expansion appliances.
- Studies including patients receiving chlorhexidine gel, varnishes or studies comparing manual vs electric toothbrushes where CHX mouthwashes were used complementarily.
- Pre-clinical studies/abstracts/letters to editors/narrative reviews.
- Insufficient/unclear information preventing data extraction.

### Search Strategy

Search strategies were developed in detail and were appropriately revised by the last author (DK) for each database, considering the differences in controlled vocabulary and syntax rules. No publication date or language restrictions were applied.

#### Electronic search

On December 7th, 2021 we updated and searched the following electronic databases to find reports of relevant published studies:

- The Cochrane Central Register of Controlled Trials (CENTRAL) (up to December 7th, 2021)
- MEDLINE (PubMed) (up to December 7th, 2021)
- Ovid MEDLINE (In-Process & Other Non-Indexed Citations, up to December7th, 2021)
- Ovid EMBASE (up to December 7th, 2021)
- LILACS (up to December 7th, 2021).

The search strategy of all databases is shown in the appendix.

#### Unpublished literature search

In order to identify more potential articles for inclusion, grey literature and possible ongoing trials were searched in the register of clinical studies hosted by the US National Institutes of Health (www.clinicaltrials.gov), the multidisciplinary European database (www.opengrey.eu), the National Research Register, and Pro-Quest Dissertation Abstracts and Thesis databases (https://about.proquest.com).

#### Manual search

Experts in the field were contacted in order to find additional literature that might be relevant. The reference lists of all eligible studies and other published systematic reviews were hand searched to identify further eligible studies. No publication time or language restrictions were applied.

### Study Selection

Study selection was performed independently and in duplicate by the first two authors of the review (IK, EK), who were not blinded to the identity of the study, their institutions, or their study results. Study selection procedure consisted of title reading, abstract reading and full-text reading stages. After exclusion of ineligible studies, a full report of eligible publications was obtained and assessed independently. Discussion and consultation with the third author of the review resolved any disagreements (KS). A record of all decisions on study identification was kept.

### Data Collection

The first two authors (IK, EK) performed data extraction independently and in duplicate. Disagreements were resolved by discussion with the last author (DK). Special Excel collection forms were used to record important information. Data collection was piloted in four randomly included papers between the two first authors. The following data were collected: author/year of study, design of study, number/age/gender of patients recruited, control group, observation period (follow-up of patients), type of mouthwash, outcome assessed, method of outcome assessment, measure of outcome, results arising from comparisons concerning CHX mouthrinse application, conclusions about the effect of CHX mouthwash.

If stated, the sources of funding, trial registration, and publishing of the trial’s protocol recorded. This information was used to aid assessment of heterogeneity and the external validity of the included studies. In case of missing data, attempts were made to contact the corresponding author.

### Quality Assessment

The methodological quality of all included studies was assessed by the first two review authors (IK, EK), independently and in duplicate. The Revised Cochrane risk-of-bias tool (ROB 2) for the randomised trials was used for this purpose.^45^ The overall quality of evidence was rated using the Grades of Recommendations, Assessment, Development and Evaluation (GRADE) approach.^25^ Concerns were resolved by discussion with the last author (DK).

### Data Analysis

Meta-analyses were conducted with included studies reporting similar interventions and comparable outcomes in a homogeneous population, i.e. in the case of limited heterogeneity. For continuous variables, mean differences and standard deviations were used to summarise the data from each study. Mean differences and 95% Cl were calculated across studies. The inverse variance statistical method was applied with a random effects analysis method. Estimates and their standard errors were entered directly into RevMan (Review Manager [computer program], version 5.4, The Cochrane Collaboration, 2020) under the ‘Generic inverse variance’ outcome. Random effects (DerSimonian and Laird) meta-analysis, along with assessment of heterogeneity, were undertaken.

### Heterogeneity

Methodological and clinical heterogeneity were evaluated by examining the study characteristics, the similarity of participant characteristics, the interventions and the study outcomes, as specified in the above-mentioned inclusion criteria for candidate studies for this systematic review. Statistical heterogeneity was assessed using a Chi^2^ test and the I^2^ statistic.

### Assessment of Reporting Bias

Reporting biases arise when the reporting of research findings is affected by the nature or direction of the findings themselves.^49^ The possibility of reporting biases including publication bias, multiple (duplicate reports) publication bias and language bias, in this review was reduced by conducting an accurate and sensitive search of multiple sources with no restriction on language and by searching for ongoing trials. In the presence of more than 10 studies in a meta-analysis, the possible presence of publication bias would have been investigated for the primary outcome.

### Subgroup Analyses/Sensitivity Analysis

Subgroup analyses based on study characteristics or sensitivity analysis based on risk of bias were not conducted, as no sufficient data existed.

### Unit of Analysis Issues

We anticipated that some of the included studies presented data from repeated observations on participants, which could lead to unit-of-analysis errors. Thus, we followed the advice provided in section 9.3.4 of the Cochrane Handbook for Systematic Reviews of Interventions:^45^ we would either define several outcomes to reflect short- and long-term observation periods, based on different time periods, and perform separate analyses, or we would select a specific time point and analyse only data at this time for studies in which it is presented.

## Results

### Study Selection

**Fig 1:**
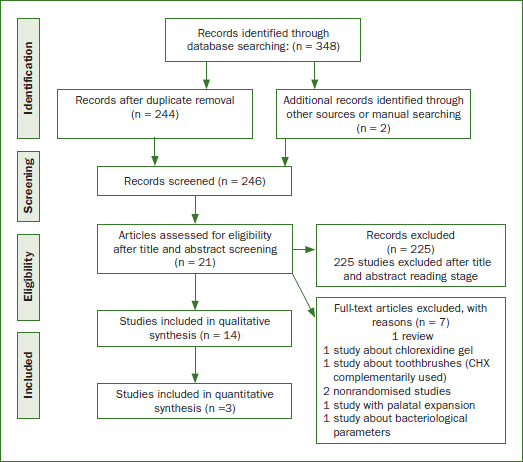
Flow diagram of studies.

Figure 1 shows the record flow of the reviewing process. Initially 348 studies were identified through database searching and 2 additional records through other sources or hand-searching. We excluded 104 as duplicates and 225 more on the basis of their title and abstract. From the 21 records that remained and were assessed as full text, 7 studies were excluded, leaving a total of 14 studies that were included in the systematic review.

### Study Characteristics

The main objective, characteristics, and outcomes of the included studies are presented in Table 1. In total, 602 patients were examined with the sample size varying from 12 to 85 participants with age ranging between 11 and 35 years. In all studies a 0.12% or a 0.2% CHX mouthwash was used, except for two studies in which a 0.06% CHX solution was used and one study in which the content of CHX solution is not mentioned. Treatment duration varied from 1 day to 3 months and the follow-up period varied from 1 min to almost 5 months. The control group used either a placebo mouthwash or a choice from a variety of mouthwashes, such as sterile isotonic saline, aloe vera, chlorine dioxide, *Matricaria chamomilla* mouthwash, isotonic saline with sodium chloride, propolis or probiotic mouthwash, herbal antiseptic mouth rinse (Persica), *Zingiber officinale* essential oil, neem mouthwash, combined mouthrinse containing chlorhexidine digluconate 0.06% and sodium fluoride 0.05%, NaF mouthrinse containing sodium fluoride 0.05% or even chlorhexidine anti-discolouration system. The effect of the mouthwashes used in treating gingivitis during treatment with fixed orthodontic appliances was assessed by means of plaque index (PI), gingival index (GI), periodontal index (CPI), oral hygiene index-simplified (OHI-S), probing depth (PD), papilla bleeding index, hyperplastic index (HI) and bonded bracket index.

**Table 1:** Main objectives, characteristics and outcomes of the included studies

Author (year) Study design	Observation period Follow-up	Inclusion criteria	Chlorhexidine (test group)		Control group	
			Intervention	Participants	Intervention	Participants
Dehghani et al (2019) RCT	Baseline 22 d	Good health status, fixed orthodontic appliances present, age range: 15–35 years, mild to moderate gingivitis present	15 ml CHX 0.12% for 1 min, twice/day	N = 19 Age: not specified Gender: not specified Mean age (N_total_ = 37): 19.86±4.19 years Gender (N_total_): 27 F, 10 M	15 ml propolis 1% mouthwash for 1 min, twice/day	N = 18 Age: not specified Gender: not specified
Shah et al (2019) RCT	1 week 2 weeks 3 weeks 4 weeks	Full dentition (except third molars) and good/fair oral and general health	10 ml CHX 0.2% in 10 ml distilled water twice/day	N = not specified Age: not reported Gender: not reported N_total_=30	Probiotic group: probiotic mouthwash (sackets of 2 x 10^8^ CFU/g in distilled water) twice/day Control group: no intervention	N_Prob_ = not specified Age: not reported Gender: not reported N_Contr_ = not specified Age: not reported Gender: not reported
Sobouti et al 2018 RCT	Baseline (immediately before OT, without mouthrinses) RCT phase of trial (mouthrinse application for 1 month): 4 months of OT 5 months of OT	Full fixed non-extraction treatment needed, lack of severe dental caries/ restorations, up to moderate dental plaque/ gingival inflammation/ periodontal condition, no systemic diseases	15 ml Orthokin mouthrinse (0.06 g CHX, 0.34 g zinc acetate ans 0.05 g NaF within 100 ml) for 30 s twice/day	N = 18 Age: not specified Gender: 11 F, 7 M Age range (N_total_ = 54): 12-21 y, mean: 14.8 y	Persica group: herbal mouthrinse (Persica, 10 drops within 2 spoons of water each day), application thrice/day Placebo group: 15 ml placebo rinse (35 ml glycerin, 35 ml distilled water, and 0.3 ml ethylene blue per 100 ml) twice/day	N_Persica_=18 Age: not specified Gender: 10 F, 8 M N_Placebo_=18 Age: not specified Gender: 10 F, 8 M
Jurišić et al 2017 RCT	Before OT (0 weeks) 6 weeks after placement (and after 14 days of rinsing) 18 weeks after placement	Good general health, no antibiotic intake or use of antibacterial rinses in the previous 3 months, no periodontal disease (no periodontal probing depth > 4 mm; bleeding on probing frequency <20%) and non-smoking	10 ml CHX 0.2% (Parodontax extra 0.2) twice/day	N = 40 (20 with metallic and 20 with ceramic brackets) Age: not specified Gender: not specified N_total_ = 80 Age range (N_total_): 11–18 y, mean: 14.2±1.4 y Gender (N_total_): 61 F, 19 M	10 ml CHX 0.2% with anti-discoloration system (CHX-ADS) twice/day	N = 40 (20 with metallic and 20 with ceramic brackets) Age: not specified Gender: not specified
Goesetal 2016 RCT	1 day 15 days	Age > 10 years, at least 20 natural teeth present, good general health, mean plaque index > 1.5, established gingivitis associated with visible plaque and concurrent with fixed orthodontic appliances, no destructive periodontal disease, at least 8 sites with bleeding on probing	15 ml CHX 0.12% mouthrinse for 1 min twice/day	N = 10 Mean age: 18.6±3.0 y Gender: not specified Gender (N_total_=30): 26 F, 4 M	Placebo group: 15 ml placebo mouthrinse twice/day *Matricaria chamomile* L. Group (MTC): 15 ml 1% MTC mouthrinse twice/day	N_Placebo_ = 10 Mean age: 21.6±6.9 years Gender: not specified NMTC = 10 Mean age: 21.5±6.0 years Gender: not specified
Yeturu et al 2015 RCT	Baseline 15 d	Age > 18 years, visible plaque and gingivitis in at least 30% of the examined teeth, fixed orthodontic treatment for at least 3 months	10 ml CHX mouthrinse for 1 min twice/day	N = 25 Mean age: 21.72±4.67 years Gender: 11 F, 14 M	Aloe vera group: 10 ml aloe vera mouthrinse for 1 min twice/day Chlorine dioxide (CD) group: 10 ml of CD for 1 min twice/day	N_Aloe_: 30 Mean age: 21.53±3.41 years Gender: 18 F, 12 M NCD: 30 Mean age: 21.70±3.01 years Gender: 16 F, 14 M
Gehlen et al 2000 RCT	Baseline (start of phases A and B, T_0_) 24 h (T_1_) 48 h (T_2_)	Right-handed patients, no pathological probing depths, fixed upper appliance with bands on molars and brackets on theother teeth for at least 6 months, same bracket type and archwire (stainless steel 016”)	Phase A: 10 ml CHX 0.2% mouthrinse (Corsodyl) twice/day for 48 h (no other OH measures) Washout phase: Odol-med-3 mouthrinse (along with regular OH) for 5 days Phase B: same with phase A	N = 5 Age: not specified Gender: not specified Mean age (N_total_=12): 14.1±1.5 y Gender (N_total_=12): 8 F, 4 M	Phase A: 10 ml fluoride mouthrinse (Odol-med-3), 10 ml twice/day for 48 h (no other OH measures) Washout phase: Odol-med-3 (along with regural OH) for 5 days Phase B: same as phase A	N = 7 Age: not specified Gender: not specified
Anderson et al 1997 RCT	Baseline 1 month 2 months 3 months Probing depth measurements only at 3 months	At least 1 banded molar/quadrant	15 ml CHX gluconate 0.12% mouthrinse (Peridex) for 30 s twice/day	N = 14 Age range: 11–15 y Gender: not reported	Placebo mouthrinse for 30 s twice/day	N = 16 Age range: 11-15 years Gender: not reported
Brightman et al 1991 RCT	Baseline 6 weeks 3 months	Age: 11-17 years, full-banded edgewise extraction treatment (4 premolars extracted, brackets on anterior teeth, bands on molars), established gingivitis, no tooth decalcification, no hypersensitivity to chlorhexidine, no medical problems or current antibiotic therapy, no anterior composites	CHX 0.12% mouthrinse (Peridex) for 30 s twice/day	N = 16 Mean age: 14.88±1.78 years Gender: not specified Gender (N_total_ = 34): 20 F, 14 M	Placebo mouthrinse for 30 s twice/day	N = 18 Mean age: 14.78±1.52 years Gender: not specified
Bauer Faria et al 2020 RCT	Baseline (before the mouthwash) 1 min after 15 min after 7th day	Average age: 19.96 years, fixed, preadjusted, and conventional orthodontic appliances in both arches from the second premolar to the second premolar for at least 6 months; good general health with low risk of periodontal disease	CHX 0.12% 10 ml for 60 s daily for 1 week	N = 31 Gender: 17 F, 14 M Average age: 19.96 years	ZOEO group: 0.5% ZOEO (*Zingiber officinale* essential oil) 10 ml for 60 s daily for 1 week Placebo group: 10 ml flavoured distilled water for 60 s daily for 1 week	N_ZOEO_ = same patients as in the test group Gender: 17 F, 14 M Average age: 19.96 years N_placebo_ = same patients as in the test group Gender: 17 F, 14 M Average age: 19.96 years
Nishad et al 2017 RCT	Baseline 30th day	Complete dentition up to the second molar, no associated comorbidities, no recent use of antibiotic or antibacterial mouthwash in recent past (1 month), no caries or demineralization	5 ml of CHX mouthwash twice/day for 30 days	N = 20 Age: 18–35 years Gender: not specified	Experimental group: 5 ml of neem mouthwash twice/day for 30 days Control group: 5 ml of distilled water twice/day for 30 days	N_experimental_ = 20 Age: 18-35 years old Gender: not specified N_control_= 20 Age: 18-35 years old Gender: not specified
Dehghani et al 2015 RCT	Baseline 22th day	Age range: 14-25 years, willingness to participate in the study, mild gingivitis, full bonded edgewise treatment with brackets on anterior teeth and premolars and bands on first molars	15 ml of CHX mouthrinse containing chlorhexidine digluconate 0.06% for 1 min twice/day for 3 weeks	N = 15 Mean age: 16.38 ± 1.45 years Gender: not specified Gender (N_total_=60): 33 F, 27 M	CHX /NaF group: 15 ml of combined mouthrinse containing chlorhexidine digluconate 0.06% and sodium fluoride 0.05% NaF group: NaF mouthrinse containing sodium fluoride 0.05% Placebo group: placebo mouthrinse	NCHX/NaF = 15 Mean age: 16.38 ± 1.45 years Gender: not specified N_NaF_ = 15 Mean age: 16.38 ± 1.45 years Gender: not specified N_Placebo_ = 15 Mean age: 16.38 ± 1.45 years Gender: not specified
Farhadian et al 2015 RCT	Baseline 2 weeks	Presence of at least 2 teeth with a HI score of 2 or higher (see below), no history of antibiotic therapy during the past sixmonths, no history of gingival problems before orthodontic therapy, no systemic conditions affecting gingival hyperplasia (e.g. pregnancy, drugs), absence of local predisposing factors (e.g. improper tooth filling, composite remnants, etc)	CHX 0.2% 15 ml for 30 s twice/day	N =18 Mean age: 18.6 ± 4.8 years Gender: not specified Gender (N_total_=72): 55 F, 17 M	Persica mouthwash: 15 drops in 15 ml of water for 20 s twice/day	Ν=18 Mean age: 18.6 ± 4.8 years Gender: not specified

RCT: randomized clinical trial; N: number; F: females; M: males; CHX: chlorhexidine; OT: orthodontic treatment; OH: oral hygiene; MB: mesiobuccally; DB: distobuccally; ML: mesiolingually; DL: distolingually. All mounthrinses were applied for the whole observation period of each study.

### Risk of Bias within Studies

The quality assessment of the included studies is presented in Table 2. Nine RCTs were judged to be at an unclear risk of bias,^3,5,13,21,37,38,42,44,53^ three studies rated as at high risk of bias,^17,19,29^ while the remaining two studies were rated as at low risk of bias.^7,12^ Regarding bias arising from the randomisation process, seven studies were at low risk, two were at high risk, and there were also some concerns for five studies regarding this domain. Moreover, six studies were considered to be at an unclear risk of bias due to lack of blinding of participants and personnel, six studies were at low risk and the last two were at high risk. Regarding bias arising the blinding of outcome assessors, there were some concerns for the majority of studies, while the remaining five studies were rated as at low risk. All fourteen studies were at low risk of bias due to missing outcome data or bias due to selective reporting.

**Table 2:** Quality assessment of the included studies

	Author/ Year	Study title	Bias arising from the randomisation process	Bias due to deviation from the intended interventions	Bias in measurement of the outcome	Bias due to missing outcome data	Bias in selection of the reporting result	Overall bias
1	Dehghani et al 2019	Effect of propolis mouthwash on plaque and gingival indices in fixed orthodontic patients	Authors’ judgement: low risk Support for judgement: use of a random component in the process of sequence generation and sequence allocation adequately concealed (similar bottles with coding by blinded person)	Authors’ judgement: low risk Support for judgement: operators and participants blinded	Authors’ judgement: low risk Support for judgement: outcome assessors blinded	Authors’ judgement: low risk Support for judgement: all outcome data available	Authors’ judgement: low risk Support for judgement: reported outcome data unlikely to have been selected	Authors’ judgement: low risk
2	Shah et al 2019	Comparative evaluation of plaque inhibitory and antimicrobial efficacy of probiotic and chlorhexidine oral rinses in orthodontic patients: a randomised clinical trial	Authors’ judgement: some concerns Support for judgement: use of block randomisation. No details provided about allocation concealment process, but there is no indication of baseline imbalance	Authors’ judgement: some concerns Support for judgement: no information provided about the blinding of operators and participants	Authors’ judgement: some concerns Support for judgement: no details provided about the blinding of outcome assessors	Authors’ judgement: low risk Support for judgement: all outcome data available	Authors’ judgement: low risk Support for judgement: reported outcome data unlikely to have been selected	Authors’ judgement: some concerns
3	Sobouti et al 2018	Effects of fixed orthodontic treatment and two new mouth rinses on gingival health: A prospective cohort followed by a single-blind placebo-controlled randomized clinical trial	Authors’ judgement: low risk Support for judgement: use of a random number table. Sequence allocation adequately concealed (uniform containers in opaque bags)	Authors’ judgement: Some concerns Support for judgement: participants blinded. Operators were blinded at the beginning, but afterwards they may have been aware of intervention	Authors’ judgement: Some concerns Support for judgement: outcome assessors were blinded at the beginning, but afterwards they may have been aware of intervention	Authors’ judgement: low risk Support for judgement: all outcome data available	Authors’ judgement: low risk Support for judgement: reported outcome data unlikely to have been selected	Authors’ judgement: some concerns
4	Jurišić et al 2017	Assessment of efficacy of two chlorhexidine mouthrinses on oral hygiene and gingival health in adolescents wearing two types of orthodontic brackets	Authors’ judgement: low risk Support for judgement: use of a random component in the sequence generation and sequence allocation adequately concealed	Authors’ judgement: high risk Support for judgement: operators aware of participants’ assigned intervention. Participants blinded	Authors’ judgement: some concerns Support for judgement: no details provided about the blinding of outcome assessors	Authors’ judgement: low risk Support for judgement: all outcome data available	Authors’ judgement: low risk Support for judgement: reported outcome data unlikely to have been selected	Authors’ judgement: high risk
5	Goes et al 2016	Clinical efficacy of a 1% *Matricaria chamomile* L. mouthwash and 0.12% chlorhexidine for gingivitis control in patients undergoing orthodontic treatment with fixed appliances	Authors’ judgement: some concerns Support for judgement: sequence generation is not described. Allocation sequence adequately concealed (identical bottles, sequentially numbered)	Authors’ judgement: low risk Support for judgement: operators and participants blinded	Authors’ judgement: some concerns Support for judgement: no details provided about the blinding of outcome assessors	Authors’ judgement: low risk Support for judgement: all outcome data available	Authors’ judgement: low risk Support for judgement: reported outcome data unlikely to have been selected	Authors’ judgement: some concerns
6	Yeturu et al 2015	Effect of *Aloe vera*, chlorine dioxide, and chlorhexidine mouth rinses on plaque and gingivitis: A randomized controlled trial	Authors’ judgement: low risk Support for judgement: use of a random component in the sequence generation and sequence allocation adequately concealed	Authors’ judgement: some concerns Support for judgement: no details provided about the blinding of operators, participants blinded	Authors’ judgement: some concerns Support for judgement: no details provided about the blinding of outcome assessors	Authors’ judgement: low risk Support for judgement: nearly all outcome data available	Authors’ judgement: low risk Support for judgement: reported outcome data unlikely to have been selected	Authors’ judgement: some concerns
7	Gehlen et al 2000	The influence of a 0.2% chlorhexidine mouthrinse on plaque regrowth in orthodontic patients	Authors’ judgement: high risk Support for judgement: no information provided about the sequence generation and the allocation concealment process	Authors’ judgement: low risk Support for judgement: operators and participants blinded	Authors’ judgement: some concerns Support for judgement: no details provided about the blinding of outcome assessors	Authors’ judgement: low risk Support for judgement: all outcome data available	Authors’ judgement: low risk Support for judgement: reported outcome data unlikely to have been selected	Authors’ judgement: high risk
8	Anderson et al 1997	Clinical effects of chlorhexidine mouthwashes in patients undergoing orthodontic treatment	Authors’ judgement: some concerns Support for judgement: presence of a random component in the sequence generation process. No details provided about allocation concealment process, but there was no indication of baseline imbalances	Authors’ judgement: some concerns Support for judgement: no details provided about the blinding of operators. Participants blinded	Authors’ judgement: some concerns Support for judgement: no details provided about the blinding of outcome assessors	Authors’ judgement: low risk Support for judgement: all outcome data available	Authors’ judgement: low risk Support for judgement: reported outcome data unlikely to have been selected	Authors’ judgement: some concerns
9	Morrow et al 1992	Clinical effect of subgingival chlorhexidine irrigation on gingivitis in adolescent orthodontic patients	Authors’ judgement: some concerns Support for judgement: use of a random component in the sequence generation (coin toss). No details provided about allocation concealment process, but there is no indication of baseline imbalances	Authors’ judgement: low risk Support for judgement: operators and participants blinded	Authors’ judgement: low risk Support for judgement: outcome assessors blinded	Authors’ judgement: low risk Support for judgement: all outcome data available	Authors’ judgement: low risk Support for judgement: reported outcome data unlikely to have been selected	Authors’ judgement: some concerns
10	Brightman et al 1991	The effects of a 0.12% chlorhexidine gluconate mouthrinse on orthodontic patients aged 11 through 17 with established gingivitis	Authors’ judgement: low risk Support for judgement: use of two randomisation tables and the mouthrinse containers were used appropriately	Authors’ judgement: low risk Support for judgement: operators and participants blinded	Authors’ judgement: low risk Support for judgement: outcome assessors blinded	Authors’ judgement: low risk Support for judgement: all outcome data available	Authors’ judgement: low risk Support for judgement: reported outcome data unlikely to have been selected	Authors’ judgement: low risk
11	Bauer Faria et al 2020	Anti -inflammatory and antimicrobial effects of Zingiber officinale mouthwash on patients with fixed orthodontic appliances	Authors’ judgement: low risk Support for judgement: all the participants received the same treatments and there was no need for a randomisation process. Sequence allocation adequately concealed (visually similar products)	Authors’ judgement: low risk Support for judgement: operators and participants blinded	Authors’ judgement: some concerns Support for judgement: no details provided about the blinding of outcome assessors.	Authors’ judgement: low risk Support for judgement: There was no information about the outcome data, but there was evidence that the result was not biased	Authors’ judgement: low risk Support for judgement: reported outcome data unlikely to have been selected	Authors’ judgement: some concerns
12	Nishad et al 2017	Impact of mouthwashes on antibacterial activity of subjects with fixed orthodontic appliances: A randomized clinical trial	Authors’ judgement: some concerns Support for judgement: Presence of a random component in the sequence generation process. No details provided about allocation concealment process, but there was no indication of baseline imbalances	Authors’ judgement: some concerns Support for judgement: no information provided about the blinding of operators and participants	Authors’ judgement: some concerns Support for judgement: no details provided about the blinding of outcome assessors	Authors’ judgement: low risk Support for judgement: all outcome data available	Authors’ judgement: low risk Support for judgement: reported outcome data unlikely to have been selected	Authors’ judgement: some concerns
13	Dehghani et al 2015	Combined chlorhexidine-sodiumfluoride mouthrinse for orthodontic patients: Clinical and microbiological study	Authors’ judgement: low risk Support for judgement: use of two random number tables. Sequence allocation adequately concealed (all of the mouthrinses had similar bottle appearance)	Authors’ judgement: some concerns Support for judgement: no details provided about the blinding of operators. Participants blinded	Authors’ judgement: low risk Support for judgement: outcome assessors blinded	Authors’ judgement: low risk Support for judgement: all outcome data available	Authors’ judgement: low risk Support for judgement: reported outcome data unlikely to have been selected	Authors’ judgement: some concerns
14	Farhadian et al 2015	Comparison of electric toothbrush, persica and chlorhexidine mouthwashes on reduction of gingival enlargement in orthodontic patients: a randomised clinical trial	Authors’ judgement: high risk Support for judgement: use of a random numbers table. No details provided about allocation concealment processed. There was the possibility of baseline imbalances	Authors’ judgement: high risk Support for judgement: both participants and operators aware of intervention	Authors’ judgement: low risk Support for judgement: outcome assessors blinded	Authors’ judgement: low risk Support for judgement: There was no information about the outcome data, but there was evidence that the result was not biased	Authors’ judgement: low risk Support for judgement: reported outcome data unlikely to have been selected	Authors’ judgement: high risk

### Study Outcomes

The reported outcomes of the included studies are presented in Table 3. It was apparent that statistically significant differences were revealed concerning plaque index, gingival index, gingival bleeding index, papilla bleeding index, bonded bracket index, hyperplastic index and probing depth scores between CHX group and control groups, especially in the first weeks of observation; moreover, CHX was found to reduce plaque accumulation and gingival inflammation more effectively than the placebo solution did.^3,7,21,44^ In the study by Dehghani et al,^13^ there were similar effects between the CHX group and the group which used a combined mouthrinse with chlorhexidine digluconate and sodium fluoride (CHX/NaF) and between the placebo group and the group which used a NaF mouthrinse with sodium fluoride (NaF) on plaque, gingival and bleeding index. However, only the CHX and the CHX/NaF groups showed statistically significant reductions of the above indices.^13^ Two other studies with CHX application in the test group, and propolis^12^ and probiotic^41^ solutions, respectively, as the control group, demonstrated similar effectiveness regarding gingival health and plaque regrowth between groups, and all succeeded in reducing the mean measured index scores. Gehlen et al^19^ compared the 0.2% CHX mouthrinse (Corsodyl) to a fluoride solution (Odol-med-3), concluding that Corsodyl application led to greater reduction in plaque accumulation and gingival inflammation. Furthermore, the study of Jurišić et al^29^ found similar effects on gingival condition and oral hygiene status using ceramic or metallic brackets alone, and CHX and CHX with antidiscoloration system alone. Potential differences between a herbal mouthwash containing alcohol extract and fluoride (Persica) and CHX were investigated in two studies, with results showing that both products were equally effective in reducing gingival bleeding and controlling plaque accumulation.^17,44^ A study by Yeturu et al^53^ compared the chlorhexidine group to two groups – either chlorine dioxide or *Aloe vera* application – and found no differences between chlorhexidine and chlorine-dioxide effectiveness. However, chlorhexidine was proved to be more effective than *Aloe vera*.^53^ In the study by Goes et al,^21^ the plaque index and the gingival bleeding index scores did not differ statistically significantly between the CHX group and the *Matricaria chamomilla* (MTC). Similarly, in the study by Morrow et al,^37^ no statistically significant differences were identified between CHX and the sterile isotonic saline group for any of the outcomes measured (plaque index, probing depth, papilla bleeding index). Moreover, the study by Nishad et al^38^ found similar reductions of plaque and gingival indices after using CHX or neem mouthwash, in contrast with the use of distilled water, which demonstrated no statistically significant reduction of the above indices. Finally, Bauer Faria et al^5^ concluded that both CHX and *Zingiber officinale* essential oil had similar effectiveness in the improvement of bonded bracket index and gingival bleeding.

**Table 3:** Reported outcomes of the included studies

Author/year Study design	Outcome assessed	Measure of outcome/method of outcome assessment	Results	Conclusions
Dehghani et al 2019 RCT	1. Gingival condition 2. Plaque accumulation	Mean score of the following indices: 1a. Gingival Index 1b. Periodontal status Index 2. Plaque Index Measurements at Ramfjord teeth	1. No statistically significant difference between groups. 2. No statistically significant difference between groups at 22 days	Similar effectiveness of CHX and propolis
Shah et al 2019 RCT	1. Gingival condition 2. Plaque accumulation	Mean score of the following indices: 1. Gingival Index measured DB, B, MB, L 2. Plaque Index measured DB, B, MB, L Measurements at teeth 16, 12, 24, 36, 32 and 44	1. Lower values in CHX and probiotics groups than in control group 2. Lower values in CHX* and probiotics groups* than in control group	Similar effectiveness of CHX and probiotics
Sobouti et al 2018 RCT	1. Gingival condition 2. Plaque accumulation T_1_: 4 months of OT, start of mouthrinse application T_2_: 5 months of OT, 1 month after T_1_)	Mean score of the following indices: 1a. Gingival Index 1b. Gingival Bleeding Index 1c. Probing depth 2. Plaque Index (PI, O’Leary) PI measurements at all teeth. Remaining measurements at Ramfjord teeth	Comparisons between T_2_ and T_1_: 1a. Lower values in Persica group than in placebo.* 1b. Lower values in Persica** and Orthokin group* than in PLC. 1c. Lower values in Orthokin group than in PLC group.* 2. Lower values in Persica** and Orthokin group*** than in PLC.	Reduction in plaque accumulation and gingival bleeding using either Persica or Orthokin
Jurišić et al 2017 RCT	1. Gingival condition 2. Oral hygiene status	Mean score of the following indices: 1. Gingival Index measured at all bonded teeth 2. Oral hygiene Index-simplified measured at 16 B, 26 B, 36 L, 46 L, 11 B and 31 B	1. Lower values in CB group at 6 and 18 weeks, where CHX-ADS was used.* 2. No statistically significant difference between groups.	Similar effects on gingival condition and oral hygiene status using ceramic and metallic brackets alone, and CHX and CHX-ADS alone.
Goes et al 2016 RCT	1. Gingival condition 2. Plaque accumulation	Mean score of the following indices: 1. Gingival Bleeding Index measured M, B, D, L 2. Visible Plaque Index measured M, B, D, L Measurements at anterior teeth.	1. Higher values in PLC group than in CHX and MTC.** No statistically significant difference between CHX and MTC group. 2. Higher values in PLC group than in CHX and MTC.*** No statistically significant difference between CHX and MTC group.	Similar effectiveness of CHX and MTC.
Yeturu et al 2015 RCT	1. Gingival condition 2. Plaque accumulation	Mean score of the following indices: 1. Gingival Index 2. Plaque Index Measurements at all teeth.	1. Lower values in CHX group than in Aloe vera group* No statistically significant difference between CHX and CD group. 2. Lower values in CHX group than in Aloe vera group.* No statistically significant difference between CHX and CD group.	Similar effectiveness of CHX and CD, but CHX more effective than Aloe vera.
Gehlen et al 2000 RCT	1. Gingival condition 2. Plaque accumulation	Mean score of the following indices per phase: 1.Gingival Index measured MB, B, DB, ML, L, DL 2. Plaque Index measured MB, B, DB, ML, L, DL and coronal to the bracket Measurements at all teeth.	1. Mean values lower in CHX group at T2.* 2. Mean values lower in CHX group at T1*** and T2.***	Reduction in plaque regrowth and gingivitis after using Cordosyl 0.2% for 2 days.
Anderson et al 1997 RCT	1. Gingival condition 2. Plaque accumulation	Mean score of the following indices: 1a. Gingival Index 1b. Probing depth measured DB, B, MB, DL, L, ML 2a. Plaque Index measured M, B, D, L 2b. Retention Index measured M, B, D, L Measurements at all teeth.	1a. No statistically significant difference at 1 and 2 months. Higher values in PLC group at 3 months.* 1b. Higher values in PLC group at 3 months*, except for the mid L areas (no statistically significant difference). 2a. No statistically significant difference at 1 and 2 months. Lower values in CHX group at 3 months. 2b. Lower values at DB and MB areas in PLC group at 1 and 2 months. No statistically significant difference at 3 months.	Reduction in plaque accumulation and gingival inflammation after using Peridex for 3 months.
Morrow et al 1992 RCT	1. Gingival condition 2. Plaque accumulation	Mean score of the following indices: 1a. Papilla bleeding index 1b. Probing depth 2. Plaque Index Measurements at the 4 interdental papilla sites on each first molar.	1a. No statistically significant difference 1b. No statistically significant difference 2. No statistically significant difference	No difference in gingival condition and plaque accumulation between groups.
Brightman et al 1991 RCT	1. Gingival condition 2. Plaque accumulation	1a. Gingival Index measured MB, B, DB, L. Mean Gingival Index score 1b. Eastman Interdental Bleeding Index measured M Gingival Bleeding Index score (%) 2. Plaque Index measured MB, B, DB, L Mean Plaque Index score Measurements on the 6 Ramfjord teeth (including 45, if 44 was extracted).	1a. No statistically significant difference at 6 weeks. Lower values in CHX group at 3 months.*** 1b. No statistically significant difference at 6 weeks. 2. Lower values in CHX group at 6 weeks.** Lower values in CHX group at 3 months.***	Significant reduction in plaque accumulation and gingival inflammation after using Peridex for 3 months.
Bauer Faria et al 2020 RCT	1. Bonded bracket index. 2. Gingival condition	1. Bonded bracket Index (Cianco): summing the index of each tooth divided by the number of teeth used. 2. Bleeding on probing: The presence or absence of bleeding was noted. Measurements at maxillary and mandibular right premolars up to the left premolars.	1. Decrease after all the treatments (CHX, PLC, ZOEO).**** Lower values in CHX and PLC groups at 7 days.**** 2. Decrease after the ZOEO and CHX mouthwash.**** Lower values in ZOEO group at 7 days.****	Reduction of biofilm and gingival bleeding after CHX and ZOEO mouthwash for 1 week.
Nishad et al 2017 RCT	1. Plaque accumulation 2. Gingival condition	Mean score of the following indices: 1. Plaque index at six sites around each tooth. 2. Gingival index from interproximal to interproximal along the BL and LL aspects of the teeth Measurements at all teeth.	1. No statistically significant difference at baseline. Lower values in CHX and neem groups after 1 month.***** 2. No statistically significant difference at baseline. Lower values in CHX and neem groups after 1 month.*****	Reduction of plaque and gingival indices after using CHX or neem mouthwash for 1 month.
Dehghani et al 2015 RCT	1. Gingival condition 2. Plaque accumulation	1a. Bleeding index measured at the BL sulcus (Saxton and van der Ouderaa) 1b. Modified gingival index measured at BL marginal gingival (Lobene et al) 2. Plaque index measured at BL surface (Turesky modification of the Quigley-Hein PI) Measurements at central incisors, canines and second premolars of four quadrants.	1a. No significant difference between CHX and CHX/NaF groups. No significant difference between NaF and PLC groups. Lower values in CHX and CHX/NaF groups after 3 weeks.******* 1b. No significant difference between CHX and CHX/NaF groups. No significant difference between NaF and PLC groups. Lower values in CHX and CHX/NaF groups after 3 weeks. ******* 2. No significant difference between CHX and CHX/NaF groups. No significant difference between NaF and PLC groups. Lower values in CHX and CHX/NaF groups after 3 weeks.*******	Significantly improved oral hygiene status after using CHX or CHX/Naf mouthrinse for 3 weeks.
Farhadian et al 2015 RCT	1. Gingiva condition 2. Plaque accumulation 3. Constructed hyperplastic index	1a. Bleeding on probing (BOP) index examined on the facial aspect (M, MD, D) and presented by percentage. 1b. Gingival index (GI) examined on the facial aspect (Loe and Sillness). 2. O’Leary’s plaque index (PI) was examined on the facial aspect (M,MD, D) and presented as percentage. 3. The constructed hyperplastic index (HI) was measured visually. Measurements at all teeth.	Both groups statistically similar at baseline for all variables. 1a. No significant difference between groups. Significant reduction in both groups after 2 weeks.******** 1b. No significant difference between groups. Significant reduction in both groups after 2 weeks.******** 2. No significant difference between groups. Significant reduction in both groups after 2 weeks.******** 3. Significant improvement in CHX group after 2 weeks.*******	Improvement of gingival conditions after using CHX or Persica mouthwash for 2 weeks. None of them could reduce gingival enlargement to the clinically acceptable level of health.

RCT: randomised clinical trial; Ramfjord teeth: the 6 teeth recommended by Ramfjord (16, 21, 24, 36, 41, 44); CHX: chlorhexidine; DB: distobuccally; B: midbuccally; MB: mesiobuccally; L: midlingually; OT: orthodontic treatment; M: mesially; D: distally; MTC: *Matricaria chamomilla*; CD: chlorine dioxide; ML: mesiolingually; DL: distolingually; CB: ceramic brackets; CHX-ADS: chlorhexidine with antidiscoloration system; ZOEO: *Zingiber officinale* essential oil; PI: plaque index; BL: buccal; LL: lingual; MD: middle; CHX/NaF: combined mouthrinse with chlorhexidine digluconate and sodium fluoride; NaF: NaF mouthrinse with sodium fluoride; PLC: placebo. * p = 0.05; ** p = 0.01; *** p = 0.001; ****p<0.005; *****p = 0.002; ******p = 0.032; *******p<0.001; ********p<0.05.

### Quantitave Analysis

**Fig 2:**
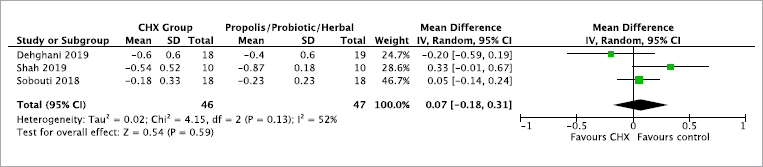
Forest plot for the comparison of gingival index at 3 or 4 weeks of intervention.

Three studies were deemed eligible for inclusion in a meta-analysis.^12,42,44^ No statistically significant difference was detected between CHX and mouthwashes with propolis/probiotics/herbs in terms of the gingival index at 3 to 4 weeks (mean difference 0.07, 95% CI: -0.18, 0.31, p = 0.59). It must be noted, however, that 2 out of the 3 studies were rated at high risk of bias,^12,42^ and thus these results should be interpreted with some caution (Fig 2). The overall quality of evidence according to the GRADE system was rated as low for probing depth (PD), hyperplastic index (HI) and bonded bracket index, or very low for the plaque index (PI), gingival index (GI), gingival bleeding index (GBI) and oral hygiene index-simplified (OHI-S) (Table 4).

**Table 4:** Summary of findings according to the GRADE approach

Outcomes	Quality of the evidence (GRADE)	No. of participants (studies)
Plaque indices (PI)	⊕OOO Very low^a^, due to inconsistency and indirectness	491 (12)
Gingival indices (GI)	⊕OOO Very low^a^, due to inconsistency and indirectness	548 (12)
Gingival bleeding index (GBI)	⊕OOO Very low^a^, due to inconsistency and indirectness	215 (6)
Oral hygiene index-simplified (OHI-S)	⊕OOO Very low^b^, due to indirectness	80 (1)
Probing depth (PD)	⊕⊕OO Low^a^, due to inconsistency	107 (3)
Hyperplastic index (HI)	⊕⊕OO Low^b^	36 (1)
Bonded bracket index	⊕⊕⊕O Moderate^c^	31 (1)

Population: orthodontic patients of any age and sex with fixed orthodontic appliances. Intervention: chlorhexidine mouthwash regardless of chlorhexidine content (%). Comparisons: any control mouthwash was accepted, including placebo solutions. ^a^Downgraded by one level for bias due to serious risk of bias for the included randomised studies. ^b^Downgraded by two levels for bias due to very serious risk of bias for the included randomised study. ^c^Downgraded by one level for bias due to serious risk of bias for the included randomised study.

## Discussion

This review provides updated information and evaluates the effectiveness of chlorhexidine mouthwash in gingivitis control of patients during the orthodontic treatment with fixed appliances, while it also constitutes the only systematic review in the literature. The results indicate that the use of mouthrinses statistically significantly reduces plaque and inflammation levels. Because of the reduced amount of plaque retention and subsequent reduction in gingival index values, less inflammation was recorded, and therefore decreased probing depths were also seen.

Orthodontic treatment with fixed appliances has been previously associated with plaque accumulation that causes disruption of gingival health, gingivitis and papilla bleeding due to qualitative and quantitative changes in dental biofilm.^40^

Preventive programs have been applied in order to improve plaque control, including various antimicrobial gels and mouthwashes. Fatima et al^18^ conducted a systematic review with meta-analysis to examine the effectiveness of antimicrobial gels on gingivitis during fixed orthodontic treatment. They concluded that the use of various antimicrobial agents resulted in significant improvement of gingivitis, but no significant difference in probing depths in follow-up visits as compared to a control group.

In this review, the differences in the gingival and plaque index as well as probing depths among patients using experimental and placebo or other mouthrinses indicated that a 0.12% or 0.2% chlorhexidine mouthrinse, used twice daily, succeeded in reducing plaque accumulation, gingival inflammation, gingival bleeding and probing depths. Comparisons showed that there was an immediate decrease in the amount of plaque present. Gingivitis severity showed a less significant reduction that was congruent with the reduction in the plaque index. This was associated with a greater reduction in number of bleeding sites at the 3-month period than at the 6-week period, as indicated in the study by Brightman et al.^7^ This also correlates directly with studies by Segreto et al^41^ and Grossman et al^23^ that recorded average reductions in the number of bleeding sites of 53% and 44%, respectively.

The low scores after chlorhexidine application during test phase suggested a carry-over effect of the chlorhexidine rinse.^19^ On the other hand, Cambell et al^8^ attributed the significantly lower plaque index scores at the beginning of test phases compared with the baseline scores to a possible Hawthorne effect, indicating that interventions in the frame of a study may itself alter the patient’s awareness and attitude. Also, Ainamo^2^ suggested that the use of chlorhexidine may be considered a motivating factor for patients, making them aware of the sensation of cleanliness and therefore developing their mechanical abilities for controlling plaque.

The decreased probing depths may have been related mainly to the lower levels of gingival inflammation. The reduction in probing depths seen as early as 2 weeks could also have resulted from variation in the probing technique.^4^

Although CHX is considered the gold standard for biofilm control and gingivitis treatment, in this review, there is evidence that other antimicrobial substances are of clinical relevance. In the study by Goes et al,^21^ the findings from patients receiving a solution of 1% MTC did not differ in relation to VPI or GBI from those receiving a CHX solution. Also, an important advantage of MTC is that it does not cause side-effects commonly associated with CHX, including ulcerations, burning sensation, alterations in taste and tooth staining.

Differences in the types of intervention used involved mainly a chlorhexidine mouthwash applied for a short period of time from 15 days to 3 months, but also single subgingival irrigation of a solution of chlorhexidine or sanguinarine and saline.^4,37^ Subgingival irrigation had a therapeutic effect on the established gingivitis related mainly to its mechanical effect rather than to an antimicrobial activity of the irrigant used.^4^ These findings contradict those of other studies, which reported no specific therapeutic effect after scaling and root planing combined with subgingival irrigation. Others^37^ show favourable changes in probing depth and bleeding scores after subgingival irrigation without concomitant root planing. It is possible that the mechanism of the observed improvement in gingival condition after subgingival irrigation was associated with a reduction of certain microorganisms or toxic products of plaque. or with a disruption of subgingival plaque, rather than instant killing of microorganisms.^35^

A similar effect was obtained in a 32-week period in the studies by Wennström et al^50,51^ and Van Strydonck et al,^48^ in which patients were treated with chlorhexidine and hydrogen peroxide irrigation. In this study,^50,51^ a permanent reduction in inflammatory lesion was not achieved, despite the extensive irrigation of the pockets and the good supragingival plaque control. Therefore, a prolonged observation period to allow a better estimate of the maintainance of the scores and the extinction of the lesions is required.^48^

This review has some limitations, mainly associated with the quality characteristics of the included studies and the data retrieved during the review process, which resulted in an assessment of the relatively low level of available evidence. The included studies employed several treatment durations and different follow-up periods. Moreover, the patients included in all studies were of different age groups and their gender was not universally reported. The sample size ranged from 12 to 85, which generated a strong methodological difference among studies.

All things considered, it is evident that more high-quality RCTs are needed, as the treatment of gingivitis in orthodontic patients will always comprise a crucial domain of clinical interest.

## Conclusions

There was considerable agreement among studies that chlorhexidine mouthwash successfully controls gingival inflammation and plaque accumulation, enhances the efficacy of oral hygiene measures and restricts plaque regrowth in orthodontic patients. In addition, it seems to promote a decrease in pocket depth. However, in the majority of the included studies, the above-mentioned effects are not statistically significantly different from the effects of a placebo solution. Moreover, chlorexidine mouthwash is as effective as other mouthrinses in terms of various oral health indices.

## Appendix

Search strategy

### PubMed Search

Strategy (updated 7.12.2021)

**Table:**

	Filters	Search Details	Results
1		(“gingiva”[MeSH Terms] OR “gingiva”[All Fields] OR “gingival”[All Fields] OR “gingivally”[All Fields] OR “gingivals”[All Fields] OR “gingivitis”[MeSH Terms] OR “gingivitis”[All Fields] OR “gingivitides”[All Fields]) AND “orthodont*”[All Fields]	3,783
2		“gingivitis”[Title/Abstract] AND “orthodont*”[Title/Abstract]	276
3		“gingivitis”[Title/Abstract] AND “orthodont*”[Title/Abstract] AND (“chlorhexidine”[MeSH Terms] OR “chlorhexidine”[All Fields] OR “chlorhexidin”[All Fields])	28
4	Randomized Controlled Trial	(“gingivitis”[Title/Abstract] AND “orthodont*”[Title/Abstract] AND (“chlorhexidine”[MeSH Terms] OR “chlorhexidine”[All Fields] OR “chlorhexidin”[All Fields])) AND (randomizedcontrolledtrial[Filter])	14
5		“chlorhexidine”[MeSH Terms] AND “orthodont*”[Title/Abstract]	139
6	Randomized Controlled Trial	(“chlorhexidine”[MeSH Terms] AND “orthodont*”[Title/Abstract]) AND (randomizedcontrolledtrial[Filter])	53
7		“orthodontic brackets”[MeSH Terms] AND “chlorhexidine”[MeSH Terms]	54
8	Randomized Controlled Trial	(“orthodontic brackets”[MeSH Terms] AND “chlorhexidine”[MeSH Terms]) AND (randomizedcontrolledtrial[Filter])	27

Keywords for Cochrane, LILACS, Embase: orthodontics, gingivitis, chlorhexidine
